# Commentary: A Bibliometric Review of Capsular Contracture in Breast Implant: Global Trends and Research Hotspots

**DOI:** 10.1177/22925503261453072

**Published:** 2026-05-30

**Authors:** Nina Hadzimustafic, Tyler Safran, Joshua Vorstenbosch

**Affiliations:** 1 Division of Plastic, Reconstructive, and Aesthetic Surgery, Department of Surgery, 5620 McGill University, Montreal, QC, Canada; 2 Division of Plastic and Reconstructive Surgery, Surgery Department, 54473 McGill University Health Centre, Montreal, QC, Canada

The most common complication impacting implant-based breast reconstruction and breast augmentation patients is capsular contracture (CC).^1–3^ Despite significant increase in research on various risk and mitigating factors in the past decade, the etiology of CC remains multifactorial and incompletely understood. In this article, Kumar et al perform a bibliometric analysis to identify current research trends and hotspots in CC.^4^ The study identifies 2065 English articles, demonstrating a significant research volume growth over the past decade, identifying the United States as the global leader, Harvard and Georgetown Universities as top institutions, *Plastic and Reconstructive Surgery* as the top journal, and highest impact authors by citation-per-publication.^4^ They highlight keywords with recent frequent occurrences in literature including *biofilm, acellular dermal matrix (ADM), contamination,* and *placement.*^4^

Kumar et al underscore the increasing interest in minimizing CC, where the highly cited research is occurring and by whom, and a trending focus on pre-clinical experimental studies in recent years.^4^ One major strength of the article is the summarization of where the most prolific and highest impact research in CC is occurring, while also identifying collaborative gaps—this will encourage researchers to undertake greater global collaboration. Furthermore, the summary of emerging and up-trending keywords in recent years both elucidates potential future research directions, as well as highlighting current hypotheses regarding biofilm and contamination as CC risk factors to plastic surgeons. The authors also discuss that although ADM and implant environment placement have been extensively studied in CC reduction, controversy and limitations in high level evidence remain. The authors interestingly identify that the highest impact studies in CC research are largely classified with low level of evidence. This underscores a need for randomized controlled trials and systematic reviews to aid in guideline development in CC risk mitigation and management.

Overall, the authors present an interesting overview of the landscape of CC research. The study is well constructed and summarizes the demographics and hotspots of publications about CC well. The findings are in keeping with a review done by Li et al (2024), identifying similar trends in demographics and keywords.^5^ Boyd et al (2024) also identifies similar trends in management, as well as highlights the significant need for a higher level of evidence body of research within CC.^6^ Kumar et al rightfully state the limitation of excluding non-English articles, which may have biased geographical demographics and collaborative efforts.^4^ Furthermore, discrete clinical impact of the findings is limited to identification of global collaborators, summary of ongoing gaps in knowledge, and direction toward higher level investigation of etiology and management of CC.

In conclusion, we would like to thank the authors for a thorough summary of the current body of literature on CC in breast surgery. This manuscript represents a rigorous, well executed, and well-summarized work that will guide clinicians and researchers alike toward global trends in CC risk mitigation and management.
